# Analysis of differentially expressed genes in oral epithelial cells infected with *Fusobacterium nucleatum* for revealing genes associated with oral cancer

**DOI:** 10.1111/jcmm.16142

**Published:** 2020-12-02

**Authors:** Shuwei Zhang, Chen Li, Zhiying Zhang, Yuchao Li, Qian Li, Fengxue Geng, Junchao Liu, Yaping Pan

**Affiliations:** ^1^ Department of Periodontics School and Hospital of Stomatology, China Medical University, Liaoning Provincial Key Laboratory of Oral Diseases Shenyang China; ^2^ Department of Oral Biology School and Hospital of Stomatology, China Medical University, Liaoning Provincial Key Laboratory of Oral Diseases Shenyang China

**Keywords:** *Fusobacterium nucleatum*, high‐throughput sequencing, lncRNA, malignant transformation, TAGs

## Abstract

Accumulating evidence links *Fusobacterium nucleatum* with tumorigenesis. Our previous study demonstrated that *F. nucleatum* infection can induce epithelial‐mesenchymal transition (EMT) in oral epithelial cells and elaborated a probable signal pathway involved in the induction of EMT. However, the comprehensive profiling and pathways of other candidate genes involved in *F. nucleatum* promoting malignant transformation remain largely elusive. Here, we analysed the transcriptome profile of HIOECs exposed to *F. nucleatum* infection. Totally, 3307 mRNAs (ǀLog2FCǀ >1.5) and 522 lncRNAs (ǀLog2FCǀ >1) were identified to be differentially expressed in *F. nucleatum*‐infected HIOECs compared with non‐infected HIOECs. GO and KEGG pathway analyses were performed to investigate the potential functions of the dysregulated genes. Tumour‐associated genes were integrated, and top 10 hub genes (FYN, RAF1, ATM, FOS, CREB, NCOA3, VEGFA, JAK2, CREM and ATF3) were identified by protein‐protein interaction (PPI) network, and Oncomine was used to validate hub genes' expression. LncRNA‐hub genes co‐expression network comprising 67 dysregulated lncRNAs were generated. Together, our study revealed the alteration of lncRNA and potential hub genes in oral epithelial cells in response to *F. nucleatum* infection, which may provide new insights into the shift of normal to malignant transformation initiated by oral bacterial infection.

## 1 INTRODUCTION

Periodontitis is a chronic infectious disease initiated by oral pathogenic bacterium, which not only causes local damage in oral sites, but also correlates with the pathogenesis of cancer.^1^ *Fusobacterium nucleatum* is a commensal bacterium colonized in the human oral cavity and is considered closely associated with periodontitis.^2^ In recent years, *F. nucleatum* has garnered a lot of attention in the initiation and progression of various malignancies, especially in colorectal cancer (CRC), through modulation of inflammation and tumour‐related signalling pathways.^3^ Based on a latest meta‐analysis, a consistent increase in the abundance of *F. nucleatum* in CRC tissues was observed and high abundance of *F. nucleatum* was associated with poorer overall survival.^4^ *F. nucleatum* was also correlated with oral cancers,^5^ and *F. nucleatum* levels were significantly elevated in oral squamous cell carcinoma (OSCC) tissues.^6^, ^7^ To date, several studies have shown that *F. nucleatum* infection affected cell proliferation, apoptosis, migration and invasion in gingival epithelial cells or OSCC cells.^8^, ^9^, ^10^ In our previous study, we established a cellular model of human immortalized oral epithelial cell (HIOECs) infected with *F. nucleatum*, and reported that *F. nucleatum* facilitated cell migration, functional loss of E‐cadherin, and up‐regulation of SNAI1 in both non‐cancerous and cancerous oral epithelial cells, which were considered as characteristics of epithelial‐mesenchymal transition (EMT).^11^ Furthermore, we uncovered a probable signal pathway involved in the induction of EMT by *F. nucleatum* infection.^11^ Nevertheless, EMT is a complicated process associated with tumorigenesis,^12^ and the comprehensive profiling and pathways of other novel candidate genes involved in the malignant transformation or tumorigenesis triggered by *F. nucleatum* infection remain unclarified.

Long non‐coding RNAs (lncRNAs) are classically defined as RNA transcripts larger than 200 nucleotides but lack of protein coding capacity.^13^ LncRNAs have been unravelled to exert their functions via modulating chromatin remodelling, transcription regulation, post‐transcriptional modifications and signal transduction.^14^ It has been shown that lncRNAs play crucial regulatory roles in various biological functions, including cell growth, differentiation, migration, invasion and EMT programs.^15^, ^16^, ^17^ Pioneering studies have demonstrated lncRNA dysregulation exert functions on EMT and tumour progression, such as lncRNA SNHG6,^18^ lncRNA HCP5,^19^ lncRNA ZEB1‐AS1^20^ and lncRNA TTN‐AS1.^21^ Our previous study suggested that lncRNA MIR4435‐2HG played a role in *F. nucleatum* inducing EMT.^11^ Moreover, many other lncRNAs and oncogenes may also participate in the regulation of cellular response to *F. nucleatum* infection. In this study, we aimed to comprehensively analyse the differentially expressed genes (DEGs) including lncRNAs and mRNAs from previous high‐throughput sequencing data for further investigation. To the best of our knowledge, this work is the first to evaluate the signatures of lncRNAs and tumour‐associated genes (TAGs) in the context of *F. nucleatum* contributing to EMT in oral epithelial cells, which may shed new light on the mechanism of *F. nucleatum* contributing to malignant transformation.

## 2 MATERIALS AND METHODS

### 2.1 Cell culture and *F. nucleatum* infection

HIOECs were provided by Prof. Wantao Chen from Key Laboratory of Shanghai Oral Medicine, Shanghai Jiao Tong University, and grown in defined keratinocyte‐SFM (Gibco) with growth supplement at 37°C and 5% CO2. *F. nucleatum* ATCC25586 was routinely cultured on tryptic soy broth agar plates containing 5% defibrinated sheep blood, 5 µg/mL hemin and 1 µg/mL menadione under anaerobic condition. The cellular model of HIOECs infected with *F. nucleatum* at a MOI of 100:1 was established as described previously.^11^ The non‐infected HIOECs were served as the control.

### 2.2 High‐throughput sequencing and data preprocessing

We established the cellular model of HIOECs infected with *F. nucleatum* at MOI of 100:1, and collected the total RNA from *F. nucleatum*‐infected HIOECs and the non‐infected HIOECs, and each had 3 repetitions, and thus, 6 RNA samples were submitted to analyse the global lncRNA and mRNA profiling at Genery Biotech Ltd. by high‐throughput sequencing on an Illumina Hiseq3000 platform (Illumina). Briefly, the raw data were filtered to remove low‐quality reads, and the resulting high‐quality data were aligned to the human reference genome (University of California at Santa Cruz hg19). Four online analytic tools, including CNCI (https://github.com/www‐bioinfo‐org/CNCI), CPAT (http://lilab.research.bcm.edu/cpat/index.php), CPC (http://cpc.cbi.pku.edu.cn/) and PLEK (https://sourceforge.net/projects/plek/), were used to predict the coding potential. Transcripts without coding potential were the candidate set of lncRNAs. DESeq2 software was used to obtain gene level fragments per kilo‐base of exon per million (FPKM) reads as lncRNA and mRNA expression profiles. Fold change and *P* value were calculated based on the FPKM data. LncRNAs and mRNAs with a significant fold change of ≧2 and *P* value < .05 were deemed as differentially expressed genes (DEGs). Furthermore, the *P* value was adjusted by multiple testing using the Benjamini‐Hochberg false discovery rate (FDR) procedure, and a standard threshold of 5% was selected for declaring significance.

Volcano plot filtering was performed to generate an overview of the differentially expressed (DE) lncRNAs and mRNAs with statistical significance between *F. nucleatum*‐infected and the control HIOECs. Target genes in cis and trans‐acting of DElncRNAs were predicted for further functional analyses. The cis role of lncRNAs indicated their actions on neighbouring target genes and the coding genes close to 100k upstream and downstream regions of lncRNAs were considered as the cis role target genes. On the contrary, the trans target genes of lncRNAs are located anywhere outside that range and identified by expression levels, according to Pearson's correlation coefficient (PCC ≧0.95).

### 2.3 Functional analysis of DEmRNAs and DElncRNAs altered by *F. nucleatum* infection

To gain insight into the functions and potential roles of the acquired DEmRNAs and DElncRNAs, Gene Oncology (GO) analyses including biological process (BP), cellular component (CC), molecular function (MF), and Kyoto Encyclopedia of Genes and Genomes (KEGG) pathway analyses were performed using the Database for Annotation, Visualization and Integrated Discovery (DAVID, https://david.ncifcrf.gov/). Bubble plot package of R software was used to visualize the results of the GO and KEGG analyses. *P* value < .05 was considered to indicate significant GO terms and pathways. Furthermore, several DEGs relevant to cell migration were summarized according to the functional analyses.

### 2.4 Screening of the tumour‐associated genes affected by *F. nucleatum* infection

Tumour‐associated genes (TAGs) including oncogenes and tumour suppressor genes were obtained from the ONGene database (http://ongene.bioinfo‐minzhao.org/) and TSGene database (https://bioinfo.uth.edu/TSGene/index.html), respectively. Then, the online Venn tool (https://bioinfogp.cnb.csic.es/tools/venny/index.html) was used to identify the overlapping genes among the DEmRNAs (ǀlog2FCǀ ≧1.5) and all the TAGs candidates. The overlapping genes were then subjected to hierarchical clustering by Multi Experiment Viewer (MEV, version 4.6.0) (JCVI) and functional enrichment analyses.

### 2.5 Protein‐protein interaction (PPI) network construction and identification of hub genes

The Search Tool for the Retrieval of Interacting Genes and Proteins (STRING: http://string‐db.org) was applied to evaluate known and predicted protein‐protein interactions. The TAGs identified from the previous step were mapped to STRING database to construct the PPI network with a high confidence of 0.70. Cytoscape software was used to visualize the network, and CytoHubba plugin was utilized to screen out the top 10 hub genes with the highest degree of connectivity in the network.^22^ *P* < .05 was considered to have statistical significance.

### 2.6 Construction of the interaction networks of DElncRNAs and hub genes

Interaction networks of DElncRNAs‐hub genes were constructed to explore the potential regulatory function of lncRNAs with aberrant expression (ǀlog2FCǀ ≧1 and *P* value < .05). Pearson's correlation coefficient (PCC) between DElncRNAs and the identified hub genes was calculated, and those pairs with PCC >0.990 were selected. The co‐expression network of DElncRNAs‐hub genes interactions was visualized using Cytoscape software.

### 2.7 Expression analysis of the hub genes and correlated lncRNAs from the co‐expression network

The expression analysis of the identified hub genes such as FOS, CREB1, JAK2, was conducted using Oncomine database (https://www.oncomine.org/), which provided genome‐wide expression analyses in various types of tumours and normal tissues. The hub genes were submitted to the Oncomine database, and seven data sets of OSCC were selected to explore their expressions in clinical samples. A *P* value < .05 and a fold change ≧2 were considered as the cut‐off with gene ranking in the top 10%. For each hub gene, we compared the results of cancerous with those of normal tissues. Additionally, the expressions of some lncRNAs identified in the co‐expression network were obtained from the GEPIA database (http://gepia.cancer‐pku.cn/), which integrated the data from The Cancer Genome Atlas (TCGA) and the Genotype‐Tissue Expression project, containing sequencing data of 9736 tumours and 8587 normal samples. The data set of HNSC was selected for analysis, and the cut‐off was set as ǀLog2FCǀ >1, *P* value < .05 and Jitter size 0.4.

## 3 RESULTS

### 3.1 DEGs in HIOECs in response to *F. nucleatum* infection

**FIGURE 1:**
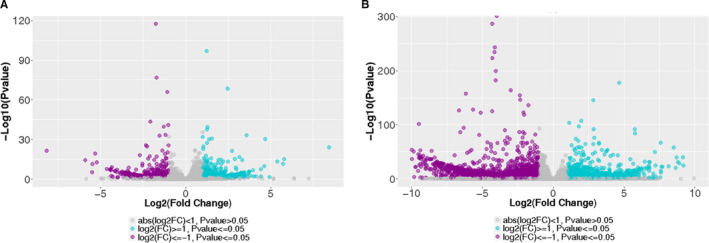
Volcano plots showing expression profiles of lncRNAs (A) and mRNAs (B) in *Fusobacterium nucleatum*‐infected HIOECs. Up‐regulated genes are marked in blue, down‐regulated genes are marked in purple. Differentially expressed genes were selected with thresholds of fold change >2 and *P* value < .05

Volcano plots were used to illustrate the general scattering of the differentially expressed lncRNAs and mRNAs between *F. nucleatum*‐infected and the non‐infected HIOECs (Figure 1). A total of 3307 mRNAs were obtained (ǀLog2FCǀ ≧1.5, *P* value < .05), of which 1261 genes were up‐regulated and 2046 genes were down‐regulated. Totally, 522 known lncRNAs with significant differential expression were identified (ǀLog2FCǀ ≧1, *P* value < .05), including 282 up‐regulated lncRNAs and 240 down‐regulated lncRNAs. The top 20 DEmRNAs and DElncRNAs were listed in Table S1.

### 3.2 GO and KEGG enrichment of DEGs

**FIGURE 2:**
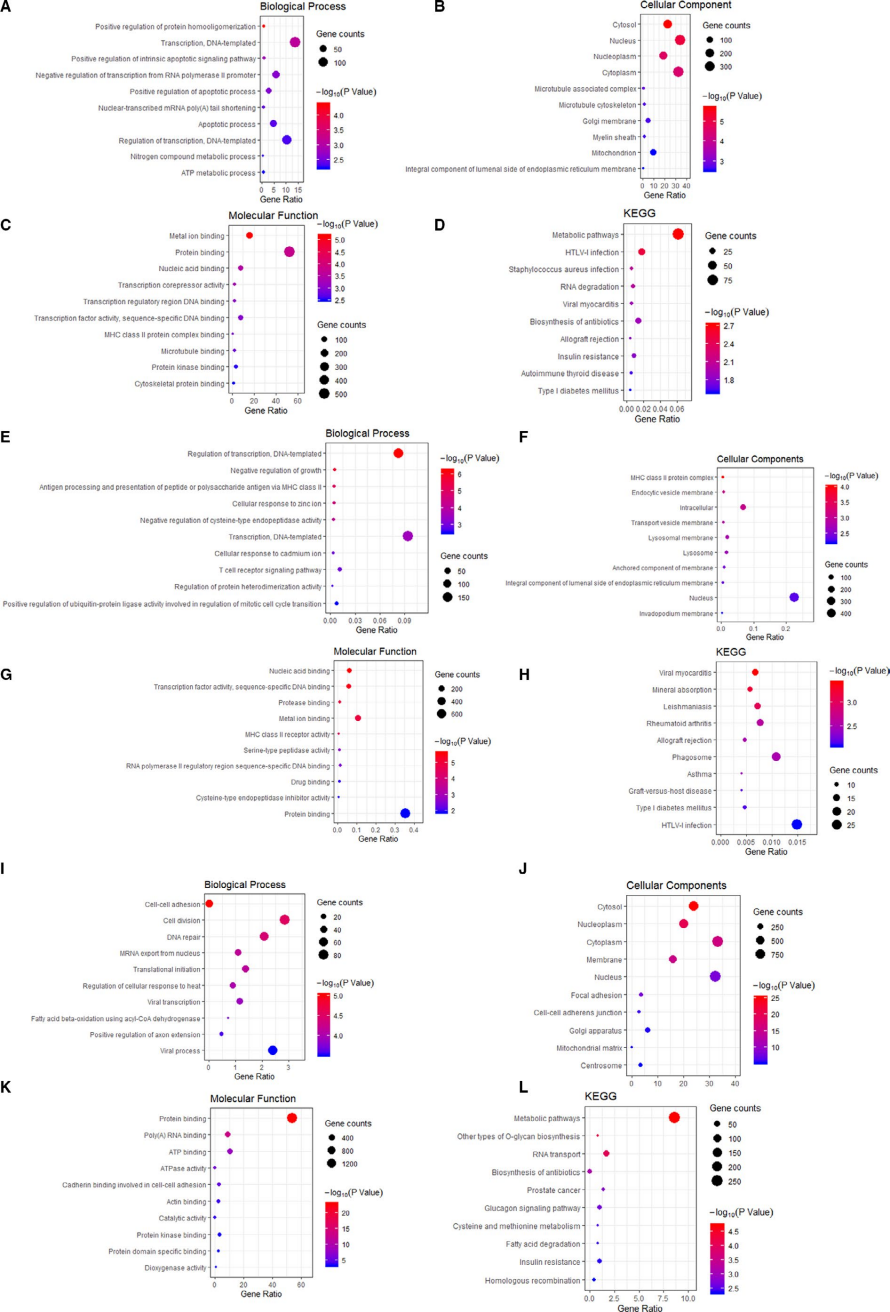
GO enrichment and KEGG pathway analysis of the DEGs. (A‐D) BP, CC, MF and KEGG items of the DEmRNA, (E‐H) BP, CC, MF and KEGG items of cis targeted genes of the DElncRNAs, (I‐L) BP, CC, MF and KEGG items of trans targeted genes of the DElncRNAs. The abscissa represents the number of genes annotated in the GO/KEGG term, the ordinate represents the GO/KEGG term, the size of the dots represents the number of genes annotated in the GO/KEGG term, and the colour of the dots represents the corrected *P* value

To decipher the biological functions of the DEmRNAs and DElncRNAs in HIOECs infected with *F. nucleatum*, GO and KEGG analysis were performed. For DEmRNAs, the DEGs were significantly enriched in biological process (BP) of DNA‐templated transcription (Figure 2A). Cellular component (CC) indicated that these genes were predominantly located in the nucleus and cytoplasm (Figure 2B). As for molecular function (MF), these genes were enriched in protein binding and metal ion binding (Figure 2C). The most significant KEGG pathways of the aberrantly expressed mRNAs were associated with metabolic pathway and HTLV‐I infection (Figure 2D), in which the genes involved in such as NAMPT, COX11, CREM and FOS, were listed in Table 1. Based on the GO enrichment analysis of the cis targeted genes of DElncRNAs (Figure 2E‐G), the most significantly enriched BP, CC and MF terms were regulation of DNA‐templated transcription, nucleus and protein binding, respectively. In terms of KEGG pathway analysis, the majority of the genes were also enriched in HTLV‐I infection pathway (Figure 2H). According to the GO and KEGG enrichment analysis of the trans targeted genes of lncRNA (Figure 2I‐L), the most significantly enriched BP, CC and MF terms were cell division and cell‐cell adhesion, cytoplasm and protein binding, respectively, and the metabolic pathways were the most remarkable pathway for enrichment. Therefore, we can speculate that the DElncRNAs have similar functions as the dysregulated mRNAs, and the lncRNAs may exert their functions via regulation of mRNAs in some way. Of note, *F. nucleatum* infection caused a variety of genetic changes involved in regulating various biological functions, and our previous study showed that *F. nucleatum* significantly promoted cell migration.^11^ In addition to MMP2/9, other genes like EGFR, PDGFA, which might participate in regulating cell migration based on the functional analysis, are listed in Table 2.

**TABLE 1:** The 2 most significant KEGG pathways and the enriched genes

Category	Term	Count	*P* value	Genes
KEGG_PATHWAY	hsa01100: Metabolic pathways	90	1.92E‐03	NAMPT, COX11, NT5C3A, NT5C3B, GGT1, PIP5K1A, DSE, UQCRQ, CKB, FDFT1, CBSL, PKM, ST6GALNAC6, TKFC, MAT1A, PRIM2, NOS3, HADH, PLCB1, PCYT2, PIGA, SELENOI, ACADM, SUCLG1, DPAGT1, ALDH3B2, DGUOK, CHPT1, PIGN, NME6, MGAT1, FLAD1, PRODH, MPST, ALDOA, CHKA, ACADSB, MGAT5B, GANAB, GALNT6, ALDOB, PPT2, CERS4, ASNS, ATP5G2, EXTL1, ATP6V1B1, POLR2C, PLPP3, POLR2B, TK2, GCH1, DGKA, MTMR3, GALNT10, B3GNT5, HAAO, ENO2, DMGDH, FUT1, BDH2, GALNT12, B4GALT4, C1GALT1, DCTD, POLR3G, DLST, PNLIPRP1, B4GALT3, ADSSL1, ACY1, UPB1, FDPS, NADK, AK5, UAP1L1, AK4, PCK2, AMPD3, GGT6, GFPT2, SMPD1, CYP2R1, GPT, HIBCH, RDH16, PLA2G4C, SCP2, PLA2G4B, PC
KEGG_PATHWAY	hsa05166:HTLV‐I infection	26	3.12E‐03	HLA‐DRB1, CREM, ITGB2, TRRAP, HLA‐DMB, FOS, XBP1, HLA‐DRB5, NFATC4, PPP3CA, PIK3R3, MYB, TCF3, APC, FZD9, SLC25A4, CREB1, FDPS, FZD3, HLA‐B, HLA‐F, GPS2, ATF3, LCK, IKBKB, HLA‐DRA

**TABLE 2:** Genes related to regulation of cell migration

Biological process	Related genes
Cell migration	EGFR, PDGFA, ELP6, EDN1, EPHA1, CDH13, CORO1A, SYNE2, ITGA6, TIAM1, ITGA5, PTK2B, F3, FGFR1OP, CARMIL1, NUMB, HAS2, LAMC2, COL1A1, PAK1, LAMB1, THBS1, CTSH, CYR61, CIB1, AJUBA, SERPINE2, SGK3, LAMA5, PLXNB1, JAG2, MINK1, PLXND1, AKT2
	LINC01006, IGFL2‐AS1, LINC01555, LINC00702, ZNF667‐AS1, LINC00511, LINC01133, LINC00460, MNX1‐AS1

### 3.3 10 genes such as FOS, CREB1 and JAK2 were identified as the hub genes from the PPI network

**FIGURE 3:**
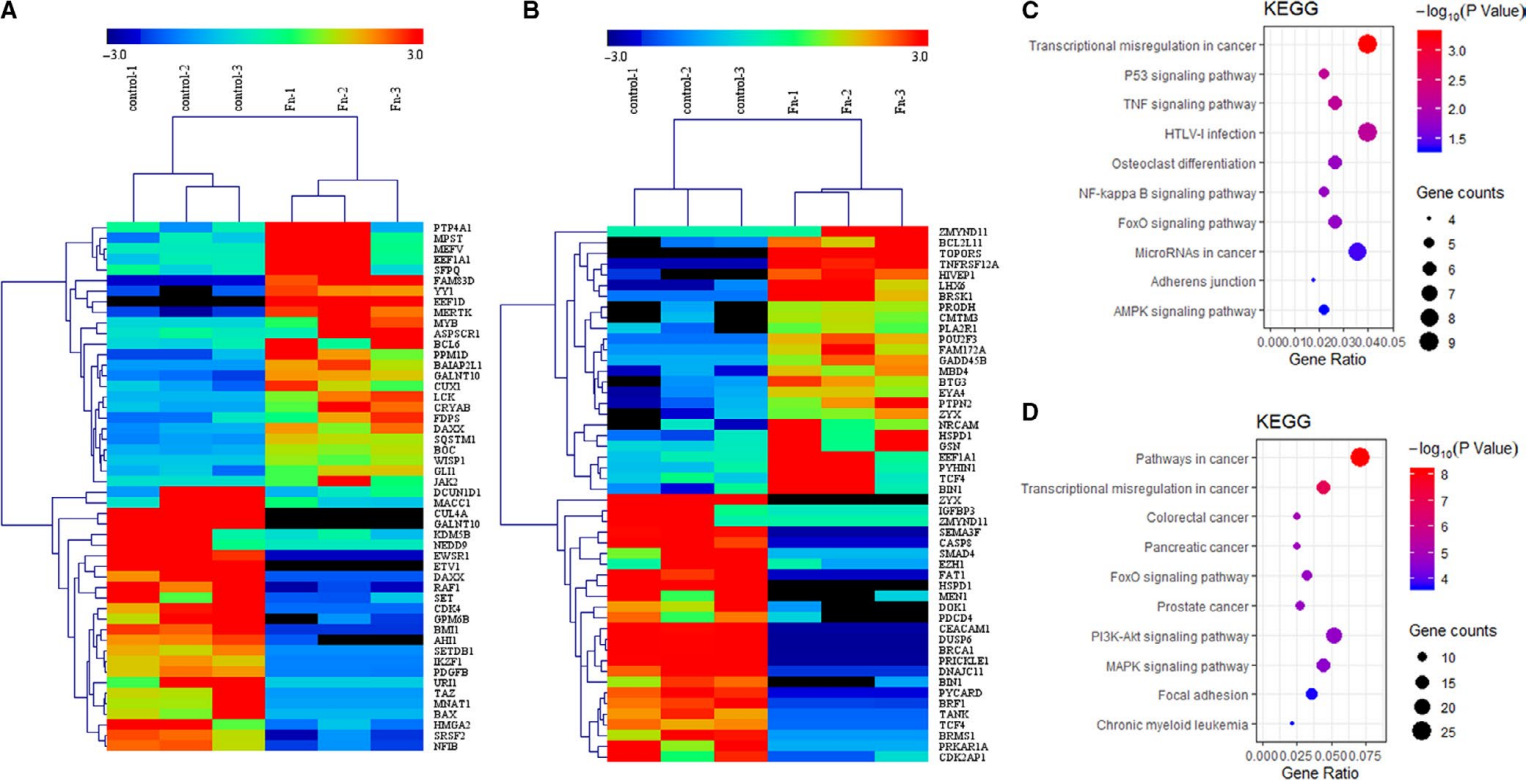
Heatmap analysis and KEGG pathway enrichment of the overlapping DEmRNAs and TAGs. (A)Top 50 genes of the overlapping part of DEGs and oncogenes. (B) Top 50 genes of the overlapping part of DEGs and tumour suppressor genes. (C) KEGG pathway analysis of the up‐regulated overlapped TAGs, (D) KEGG pathway analysis of the down‐regulated overlapped TAGs

**FIGURE 4:**
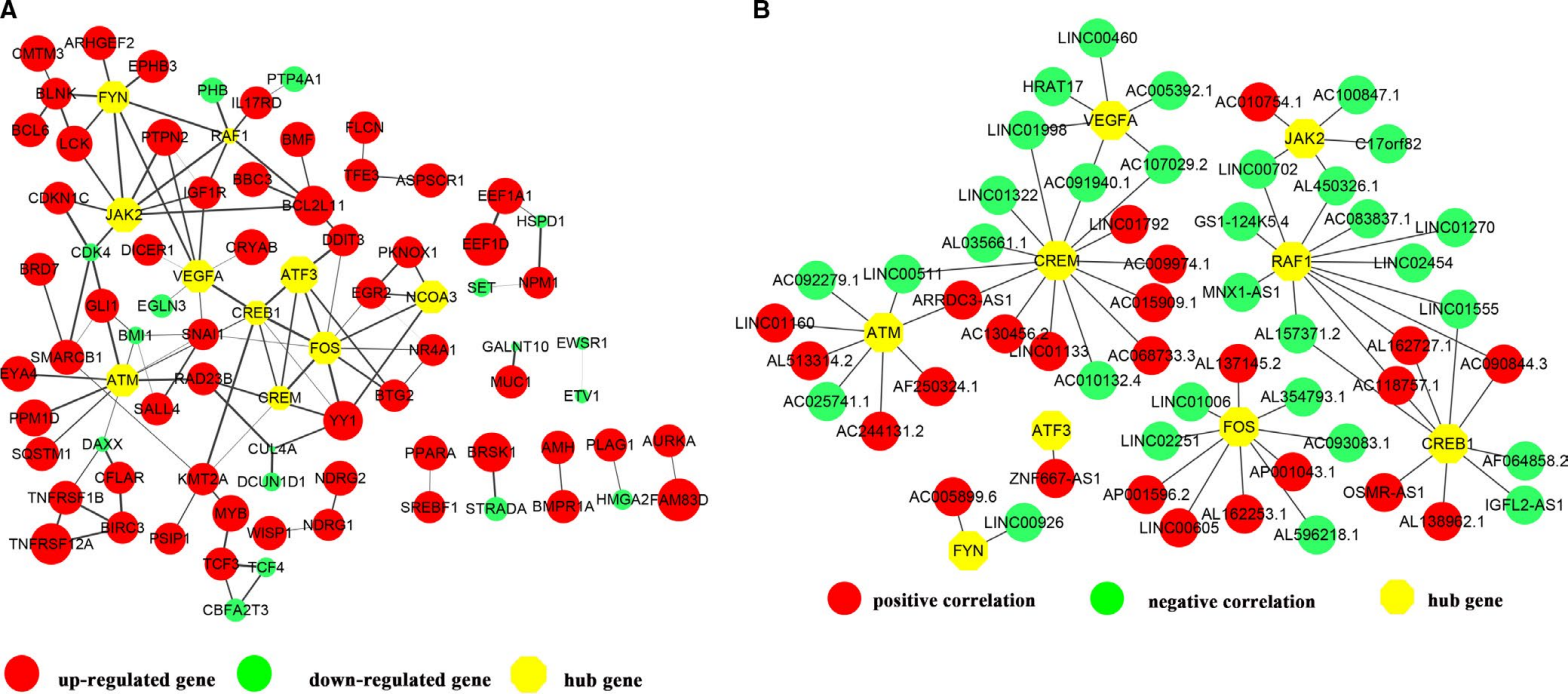
PPI analysis of the TAGs and co‐expression of the DElncRNAs and hub genes. (A) A protein‐protein interaction (PPI) network constructed by STRING was visualized with Cytoscape. 83 nodes and 106 edges were displayed. Up‐regulated genes are shown in red, whereas down‐regulated genes are shown in green. The hub genes were labelled with octagon in yellow. Larger node sizes correspond to higher fold changes of DEGs. Edges were shown in grey from light to dark according to the combined score (from low to high). (B) Co‐expression network of DElncRNAs and hub genes. The circle represents lncRNA and the octagon in yellow represents the hub genes. Red represents positive correlation and green represent negative correlation. Edges between two nodes represent interaction between lncRNAs and hub genes

Among the DEmRNAs, a total of 353 genes were screened out as TAGs, including 66 up‐regulated and 56 down‐regulated oncogenes, 86 up‐regulated and 108 down‐regulated tumour suppressor genes and 37 genes functioning as both oncogene and tumour suppressor genes (Table S2). The 50 most dysregulated genes including 25 most up‐regulated and down‐regulated were listed in a heatmap clustering analysis (Figure 3A,B). Additionally, KEGG analysis revealed that the up‐regulated TAGs were mainly enriched in transcriptional mis‐regulation in cancer and HTLV‐I infection (Figure 3C), and the down‐regulated TAGs were enriched in pathways in cancer and transcriptional mis‐regulation in cancer (Figure 3D). Then the selected TAGs were used to construct a PPI network. As shown in Figure 4A, the PPI networks of aberrantly expressed TAGs consisted of 83 nodes and 106 edges, including 62 up‐regulated gens and 20 down‐regulated genes. As emphasized with octagon in yellow, FOS (Fos Proto‐Oncogene), CREB1 (CAMP Responsive Element‐Binding Protein 1), JAK2 (Janus Kinase 2), ATF3 (Activating Transcription Factor 3), ATM (ATM Serine/Threonine Kinase), FYN (FYN Proto‐Oncogene, Src Family Tyrosine Kinase), CREM (CAMP Responsive Element Modulator), VEGFA (Vascular Endothelial Growth Factor A), RAF1(Raf‐1 Proto‐Oncogene, Serine/Threonine Kinase) and NCOA3 (Nuclear Receptor Coactivator 3) were identified as the top 10 hub genes with high degree of connectivity of the network, which were deemed to be functionally critical and immensely interconnected with other genes.

### 3.4 Co‐expression network of DElncRNAs and hub genes

To evaluate the possible regulatory functions of the DElncRNAs on the hub TAGs in the progression of malignant transformation or tumorigenesis, the co‐expression network analysis was performed. The network showed that the hub genes might be co‐regulated by different lncRNAs and one lncRNA might regulate several hub genes (Figure 4B). For instance, FOS could be regulated by LINC00605 positively and by LINC01006 negatively, whereas LINC00511 might regulate both the expressions of ATM and CREM, yet the specific mechanism needs further investigation. Meanwhile, 9 known lncRNAs (LINC01006, IGFL2‐AS1, LINC01555, LINC00702, ZNF667‐AS1, LINC00511, LINC01133, LINC00460, and MNX1‐AS1) associated with cell migration according to the available publications are also summarized in Table 2.

### 3.5 Validation of hub genes and lncRNAs in clinical samples

**FIGURE 5:**
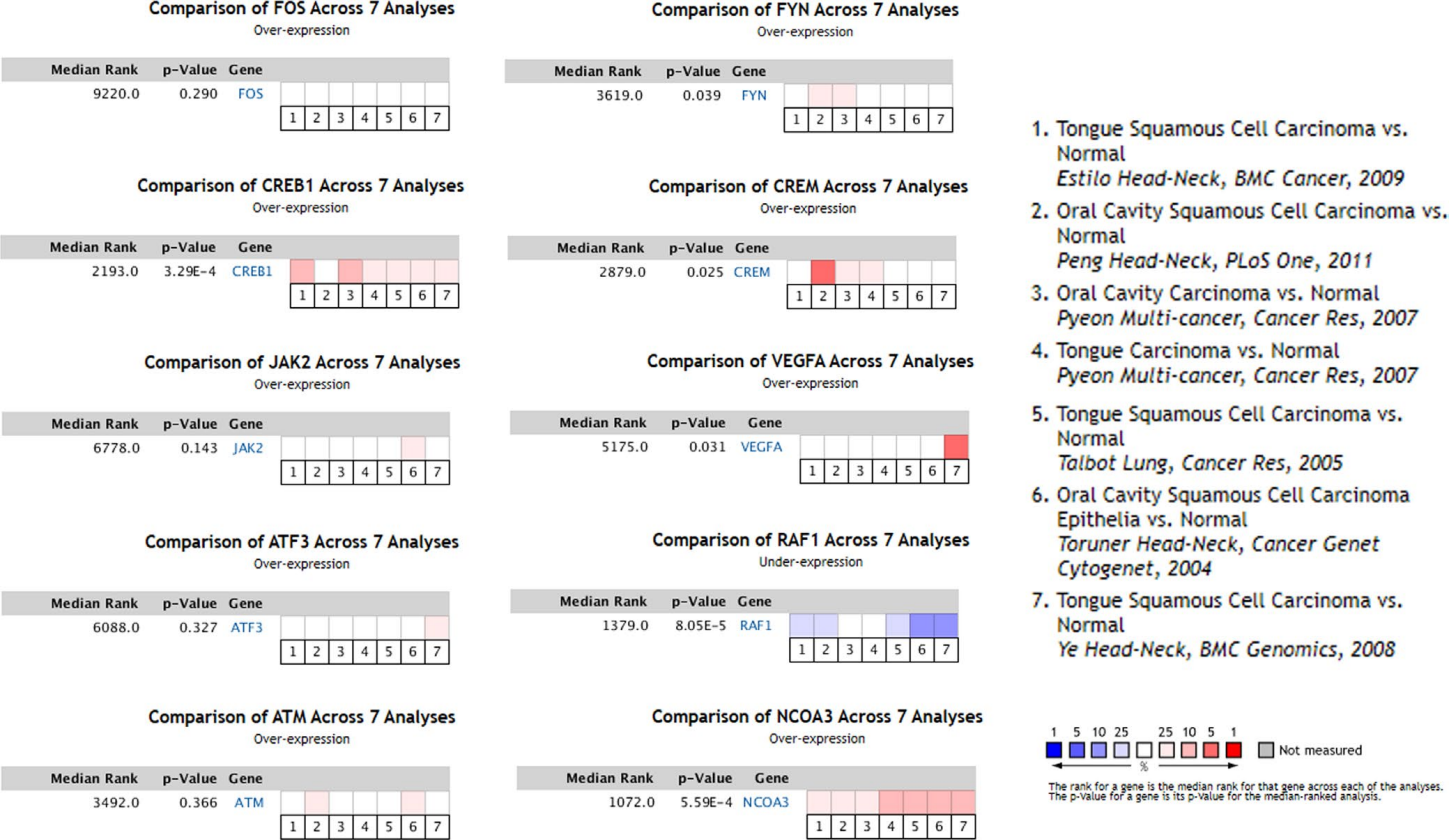
Seven OSCC data sets were selected and the expression of the hub genes was compared across the seven data sets. Normal tissues were selected as the control. Values above the average were considered overexpressed hub genes (red)

**FIGURE 6:**
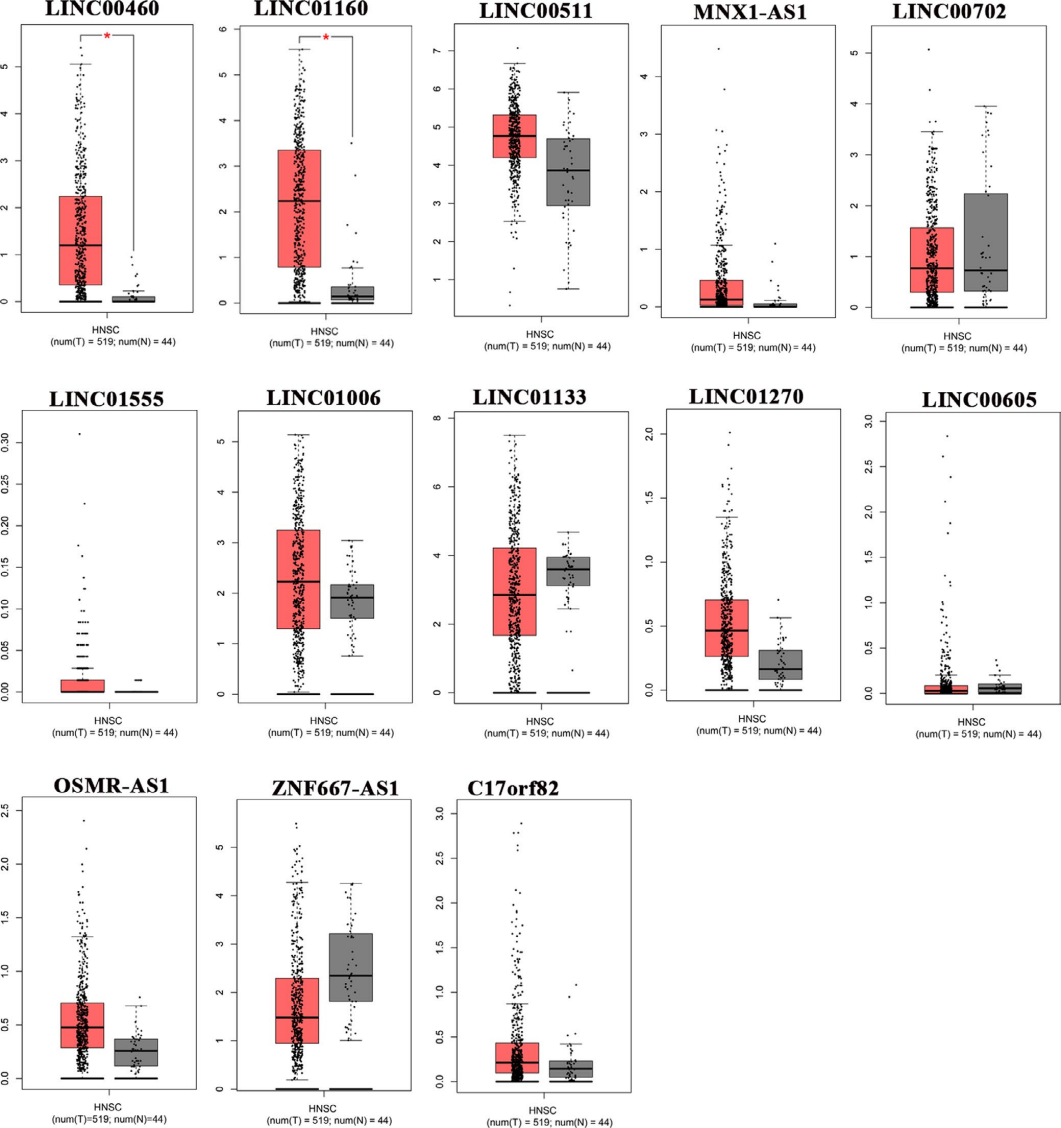
GEPIA database analysis for gene expression of several lncRNAs in head and neck cancer. The RNA expression levels of LINC00460 and LINC01160 were significantly increased in head and neck cancer (n = 519) compared to those in normal tissues (n = 44) (*P* < .05)

To determine whether the hub genes identified in HIOECs infected with *F. nucleatum* were also aberrantly expressed in oral cancer, seven data sets of clinical samples of OSCC from Oncomine database were selected to validate the expressions of the hub genes. Notably, CREB1, CREM and NCOA3 were overexpressed in more than 3 data sets, whereas RAF1 was significantly under‐expressed in 5 data sets (Figure 5). In addition, the expressions of several DElncRNAs in the co‐expression network were assayed by analysing the data set from the GEPIA database, which contains research data both from TCGA and GTEx. As shown in Figure 6, LINC00460 and LINC01160 were significantly up‐regulated in head and neck squamous cell carcinoma (HNSC) tissues compared with normal tissues (*P* < .05) Other lncRNAs including C17orf82, LINC00511, LINC00605, LINC00702, and MNX1‐AS1 were also detected in HNSC tissues, but the box plots showed no statistic difference between the HNSC and normal tissues. However, they were aberrantly expressed in other types of cancer, such as colon adenocarcinoma (COAD), oesophageal carcinoma (ESCA), pancreatic adenocarcinoma (PAAD) and rectum adenoma (READ), which have been reported to be associated with *F. nucleatum* infection.^23^, ^24^ For instance, LINC00702 were identified to be under‐expressed in both COAD and READ, MNX1‐AS1 were found to be overexpressed in COAD and READ , and LINC00511 were overexpressed in COAD, ESCA, PAAD and READ compared with their respective normal tissues (Figure S1).

## 4 DISCUSSION

*Fusobacterium nucleatum* is a well‐recognized commensal bacterium commonly localized in the human oral cavity, but also can be found in individuals with extraoral infections, including gastrointestinal and cardiovascular diseases, rheumatoid arthritis, respiratory tract infections, and pancreatic cancer.^2^, ^25^, ^26^ Extensive researches using metagenomic and transcriptomic analyses have shown that *F. nucleatum* was highly elevated in a subset of colorectal adenoma and carcinoma tissues.^27^, ^28^, ^29^ In addition, the amount of invasive *F. nucleatum* gradually increased from premalignant adenomatous lesions to carcinomas in the colorectal carcinogenesis pathway, suggesting *F. nucleatum* could be used as a novel risk factor for disease progression from adenoma to cancer.^30^, ^31^ A recent meta‐analysis showed that *Fusobacterium* was in higher abundance in oral/head and neck cancer.^32^ Compared with the non‐tumour lesions, the prevalence of *Fusobacterium* in tumour lesions increased by 6% (95% CI, 3‐9), and a 2.93 higher chance (95% CI, 1.47‐5.81) of *Fusobacterium* being present in tumour lesions,^32^ suggesting that *Fusobacterium* infection might contribute to oral/head and neck cancer. In recent years, the researchers have focused on the associations between *F. nucleatum* status and clinicopathological and molecular features, as summarized in Table 3. Although *F. nucleatum* has been detected to be enriched in OSCC, the clinical characteristics associated with *F. nucleatum* abundance were still in deficiency. Among the publications available online, only one study conducted by Yang et al analysed the correlation between the clinical features and *Fusobacteria*, concluding that increased colonization by *Fusobacteria* was positively associated with OSCC stage.^33^ However, several studies have investigated the association between clinicopathological features, including tumour location, differentiation, TNM stage and *F. nucleatum* status in colorectal cancers and oesophageal squamous cell carcinoma (ESCC), suggesting that higher *F. nucleatum* burden was not significantly associated with the tumour location and differentiation, but was associated with advanced tumour stage and poor survival. Also, *F. nucleatum* burden was observed to be associated with molecular features such as KRAS, MSI, MLH1 and CIMP status.^24^, ^34^ The discrepancy among studies may be due to different methods used for sampling and for identification of *F. nucleatum*. In addition, the sample size and demographic characteristics of the participants may also give rise to the heterogeneity. These studies provide more evidence that *F. nucleatum* is associated with tumorigenesis and could be a potential risk factor for OSCC and extraoral cancers, especially CRC.

**TABLE 3:** Studies showing the association between clinical pathological features and *Fusobacterium nucleatum* infection in cancers

Researches	Cancers	Samples	Association
Yang et al, 2018	OSCC	Oral rinse	Increased colonization by *Fusobacteria* was positively associated with OSCC and stage
Bronzato et al, 2020	Oral/head and neck cancer	A meta‐analysis of 17 publications	Increased prevalence of 6% (95% CI, 3‐9) of *Fusobacterium* in tumour lesions than in non‐tumour lesions, and a 2.93 higher chance of *Fusobacterium* being present in tumour lesions (95% CI, 1.47‐5.81)
Yamamura et al, 2019	ESCC	FFPE tissues	Higher *F. nucleatum* burdens correlates with poor recurrence free survival and poor response to neoadjuvant chemotherapy, but was not associated with age, gender, tumour location and differentiation. Higher intratumoral *F. nucleatum* levels were associated with higher T category and larger tumour size and higher TNM stage
Chen et al, 2019	CRC	FFPE tissues	No significant differences in the tumour location, differentiation, and stage were observed between Fn high and Fn‐low/negative groups. Fn high abundance was associated with lymph node, neurological invasion and vascular tumour thrombus, and shorter survival time
Mima et al, 2015	CRC	FFPE tissues	A higher amount of Fn in CRC tissues was associated with stage Ⅱ‐Ⅳ, poor differentiation, and MSI‐high, MLH1 hypermethylation and CIMP‐high status. No significant difference with tumour location was observed.
Proenca et al, 2018	Colorectal adenoma (CRA) and colorectal cancer (CRC)	Fresh tissues	Overabundance of Fn in neoplastic tissues compared to matched normal tissues was detected in CRA (51.8%) and more markedly in CRC (72.1%). KRAS mutation were more frequent in Fn‐infected CRC
Kashani et al, 2020	CRC	Biopsy samples	No statistically significant correlations were found between Fn colonization and demographic parameters, including age, diet and gender.
Mitsuhashik et al, 2015	PDAC	FFPE tissues	Tumour Fusobacterium status was not associated with tumour location, tumour size, lymph node invasion and stage, and not associated with molecular features including KRAS, MLH1 and CIMP status, but is independently associated with a worse prognosis of pancreatic cancer.

The most important carcinogenesis mechanisms of *F. nucleatum* are chronic infection, interaction of cell surface molecules of these bacteria with immune system and stromal cells, immune evasion and immune suppression.^23^ And a portion of genes dysregulated by *F. nucleatum* infection in cancers confirmed in previous studies were summarized in Table 4, such as CCL20,^25^ TNFSF9,^35^ CEACAM1.^36^ However, there might have some other unexplored regulatory genes affected by *F. nucleatum* infection in the malignant transformation. In this study, we mined the high‐throughput sequencing data obtained from a cellular model of oral epithelial cells infected by *F. nucleatum* at a MOI of 100.^11^ The function of the DEGs and cis targeted genes of differentially expressed lncRNAs were significantly enriched in biological process of DNA‐templated transcription, whereas the trans targeted genes of lncRNAs were mainly associated with cell division and cell‐cell adhesion. Then, we specifically analysed the tumour‐associated genes and identified the top 10 hub genes related to tumour progression, and confirmed that some of the hub genes were also aberrantly expressed in OSCC clinical specimens.

**TABLE 4:** Reported genes involved in *Fusobacterium nucleatum*‐associated tumours

Genes	Regulation	Role	Cancer	Reference (PMID)
CARD3 LC3‐II P62	Up Up Down	Activate the autophagy pathway and promote metastasis Autophagy	CRC	Chen et al, 2020
CREBBP	Down	Act as an oncogene and promote CRC progress	CRC	Feng et al, 2019
TNFSF9	Up	Correlated with reduced overall survival in pancreatic adenocarcinoma patients Regulate immune cell infiltration	Pancreatic adenocarcinoma (PAAD)	Wu et al, 2019
ANXA1	Up	Activate cyclinD1 and promote proliferation of CRC cells High expression of ANXA1 as a predictor of poor prognosis in CRC	CRC	Rubinstein et al, 2019
ANO1	Up	Prevent colon cancer apoptosis from oxaliplatin and 5‐FU	CRC	Lu et al, 2019
CEACAM1	Up	Fn directly interacts with CEACAM1 and suppress anti‐tumour immunity of T cells and NK cells activity	CRC	Gur et al, 2019
BIRC3	Up	Inhibit apoptosis	CRC	Zhang et al, 2019
ATG7 ULK	Up	Inhibit apoptosis and enhance chemoresistance Activate autophagy	CRC	Yu et al, 2017
BECN1	Down	Tumour BECN1 expression inversely correlated with the amount of Fn DNA	CRC	Haruki et al, 2020
CCL20	Up	Recruit immune cells to neoplastic regions Present at all stages of tumours	CRC	Ye et al, 2017

Special attention was paid to the tumour‐associated genes including oncogenes and tumour suppressor genes. Among the genes shown in Figure 4A, several hub genes such as JAK2, ATF3, ATM, NCOA3 and RAF1 were found to be involved in regulation of EMT process. The tumour‐promoting functions of the JAK2/STAT3 signalling pathway have been well recognized in various malignancies,^37^ and JAK2/STAT3 participates in promoting EMT and enhancing the stemness of OSCC.^38^ ATF3 (activating transcription factor 3) is an adaptive‐response gene in the ATF/cAMP responsive element‐binding (CREB) protein family of transcription factors and has been considered to be involved in the complex process of cellular response provoked by a wide range of intra‐ and extracellular stresses.^39^ You et al investigated the mechanism of ATF3 on cell proliferation, invasion, migration and EMT in cholangiocarcinoma, and demonstrated that ATF3 repressed EMT and cell viability in cholangiocarcinoma via p53 signalling pathway.^40^ However, the aberrant expression or regulatory function of ATF3 in OSCC or in *F. nucleatum*‐infected host cells has seldomly been reported ever. ATM (ataxia telangiectasia mutated) has been reported to trigger a cascade of signalling reactions mediating phosphorylation of Akt, GSK3ß and Snail to promote EMT in breast cancer cells.^41^ Russell et al investigated the intimate link between ATM expression and pancreatic cancer progression and demonstrated that loss of ATM accelerates pancreatic cancer formation and EMT.^42^ He et al found that ATM protein levels were higher in oral leukoplakia than in normal controls, whereas the expressions of ATM protein showed a versatile tendency in OSCC tissues, suggesting that ATM might be a candidate biomarker for diagnosis and prognosis in OSCC, as well as a possible genetic marker for early‐onset OSCC.^43^ NCOA3, also known as AIB1, is an important member of the p160 steroid receptor coactivator (SRC) family and interacts with nuclear steroid hormone receptors. NCOA3 is amplified and expressed in a wide spectrum of human malignancy diseases,^44^ and studies have demonstrated that NCOA3 promoted EMT via the PI3K/AKT signalling in gastric cancer or through SNAI1 activation in breast cancer.^45^, ^46^ However, the role of NCOA3 in the regulation of oral cancer cell EMT has not been sufficiently elaborated. RAF1 is a cytosolic serine/threonine protein kinase and plays an important role in mitogen and stress‐induced signalling responses, proliferation and cell survival.^47^ Feng et al found that knockdown of RAF1 decreased the cell migration ability and dampended EMT of HK2 cells.^48^ As a member of the Ras family, RAF1 has been reported to activate the MEK/ERK signalling cascade and regulate the EMT process.^49^

**FIGURE 7:**
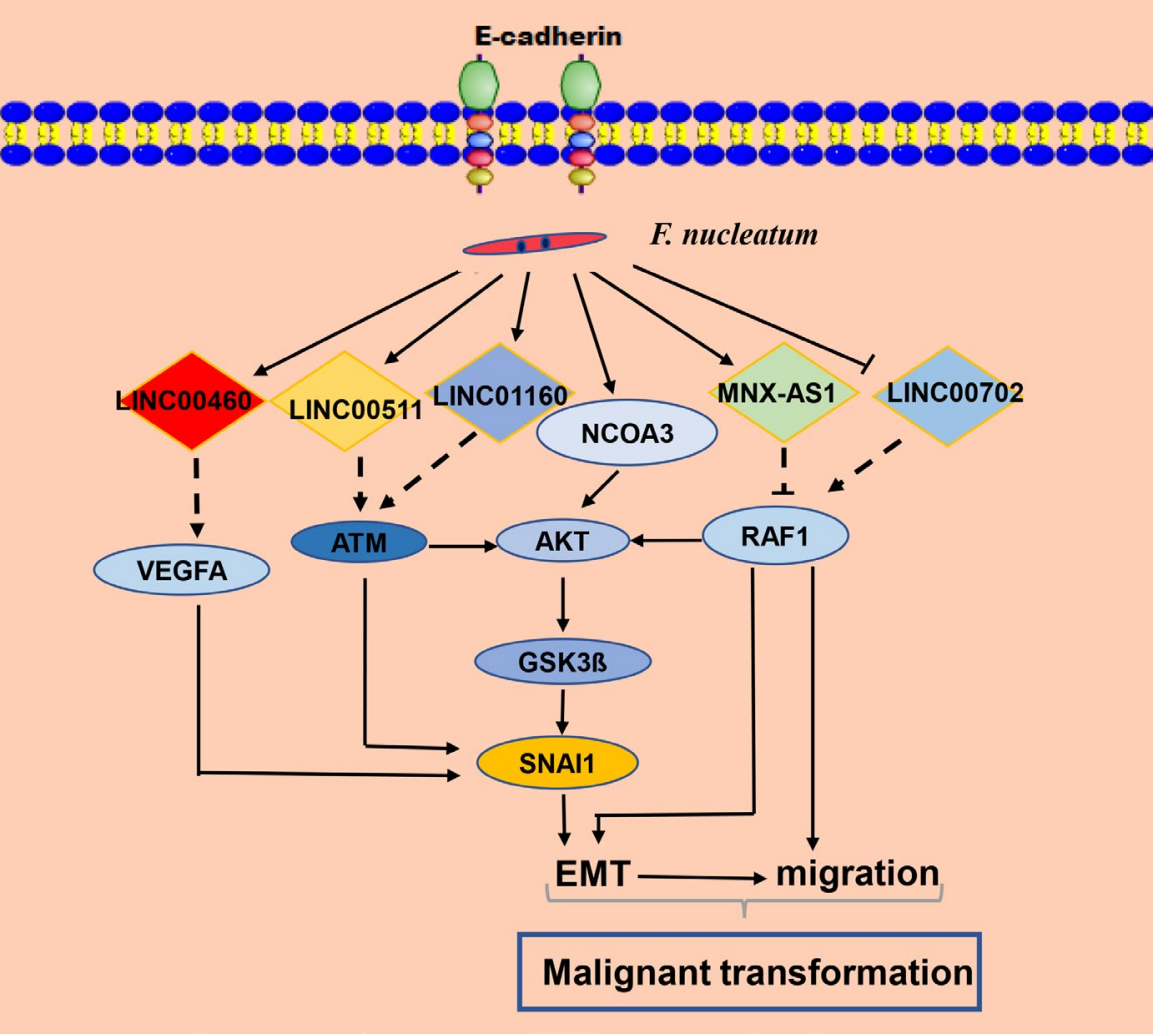
Proposed pattern diagram. According to our analysis results, LINC00460, LINC00511, LINC01160, LINC00702 and MNX1‐AS1 might act as key regulators of the hub genes involved in the malignant transformation induced by *Fusobacterium nucleatum* infection. SNAI1 was previously validated. The solid arrows between the genes indicated the proven relationship, whereas the dashed lines indicated the interaction inferred from our bioinformatic analysis that has not been experimentally verified

Except for the differentially expressed coding genes, the aberrantly expressed lncRNAs were also identified in HIOECs infected with *F. nucleatum* in our present study. Given that a number of the differentially expressed lncRNAs were unknown or rarely studied, we therefore mainly focused on the known lncRNAs. For the lncRNAs in the co‐expression network that might regulate the expression of the hub genes (Figure 4B), pioneering studies have demonstrated their dysregulation might result in the progression of EMT or tumorigenesis, such as LINC01006,^50^ LINC00511^51^ and LINC01133.^52^ Of note, LINC00460 has been unravelled to play a positive role in EMT progress in HNSCC,^53^ and LINC00460 was in a significantly higher expression level in OSCC tissues compared with the normal tissues validated by the GEPIA database (Figure 6). As shown in Figure 4B, LINC00460 was predicted to be in a co‐expression pattern with VEGFA, which has been proved to be overexpressed in aggressive OSCC.^54^ We can speculate that LINC00460 may contribute to oral malignancy through regulating VEGFA, which needs further study. MNX1‐AS1 is a natural antisense transcript of MNX1 and has been reported to act as an oncogene in a variety of cancers.^55^, ^56^, ^57^ Cheng et al demonstrated that overexpression of MNX1‐AS1 could induce EMT and activate Akt/mTOR pathway in breast cancer.^57^ Wu et al reported that MNX1‐AS1 mediated EMT of the osteosarcoma cells via activating MNX1, thereafter accelerating the progression of the osteosarcoma.^58^ However, its role in regulating oral cancer‐associated biological processes remains unclear. By comprehensively analysing the data shown in Figure 4, lncRNAs including LINC00460, LINCO00702, LINC01160 and MNX1‐AS1, and hub genes ATM, NCOA3, and VEGFA were considered to be potential candidate genes and presented in the proposed mode pattern in Figure 7.

To date, several studies have reported the promoting effects of bacteria such as *Porphyromonas gingivalis* and *Escherichia coli* on EMT process or malignant transformation.^59^, ^60^ Microbial pathogens may be considered as EMT inducers, and regulate the relevant signalling pathways. Overall, our present study identified the dysregulated lncRNAs and screened the key genes aroused by *F. nucleatum* infection and shed light on the complex regulatory networks in host cells in response to bacteria, and further experiments are needed to interrogate and validate the identified genes.

## CONFLICT OF INTEREST

The authors confirm that there are no conflicts of interest.

## AUTHOR CONTRIBUTION

**Shuwei Zhang:** Data curation (lead); Formal analysis (lead); Software (lead); Writing‐original draft (lead). **Chen Li:** Conceptualization (supporting); Writing‐review & editing (supporting). **Zhiying Zhang:** Formal analysis (supporting). **Yuchao Li:** Formal analysis (supporting). **Qian Li:** Formal analysis (supporting). **Fengxue Geng:** Data curation (supporting); Formal analysis (supporting). **Junchao Liu:** Data curation (supporting). **Yaping Pan:** Conceptualization (supporting); Formal analysis (supporting); Funding acquisition (lead); Writing‐review & editing (supporting).

## Supporting information

Fig S1

Table S1‐S2

## Data Availability

The data sets used and/or analysed during the current study are available from the corresponding author on reasonable request.
