# Supplementary figures and images for: Analysis of differentially expressed genes in oral epithelial cells infected with Fusobacterium nucleatum for revealing genes associated with oral cancer
Source: J Cell Mol Med. 2020 Dec 2;25(2):892–904. doi: 10.1111/jcmm.16142 (PMC7812288; doi:10.1111/jcmm.16142)

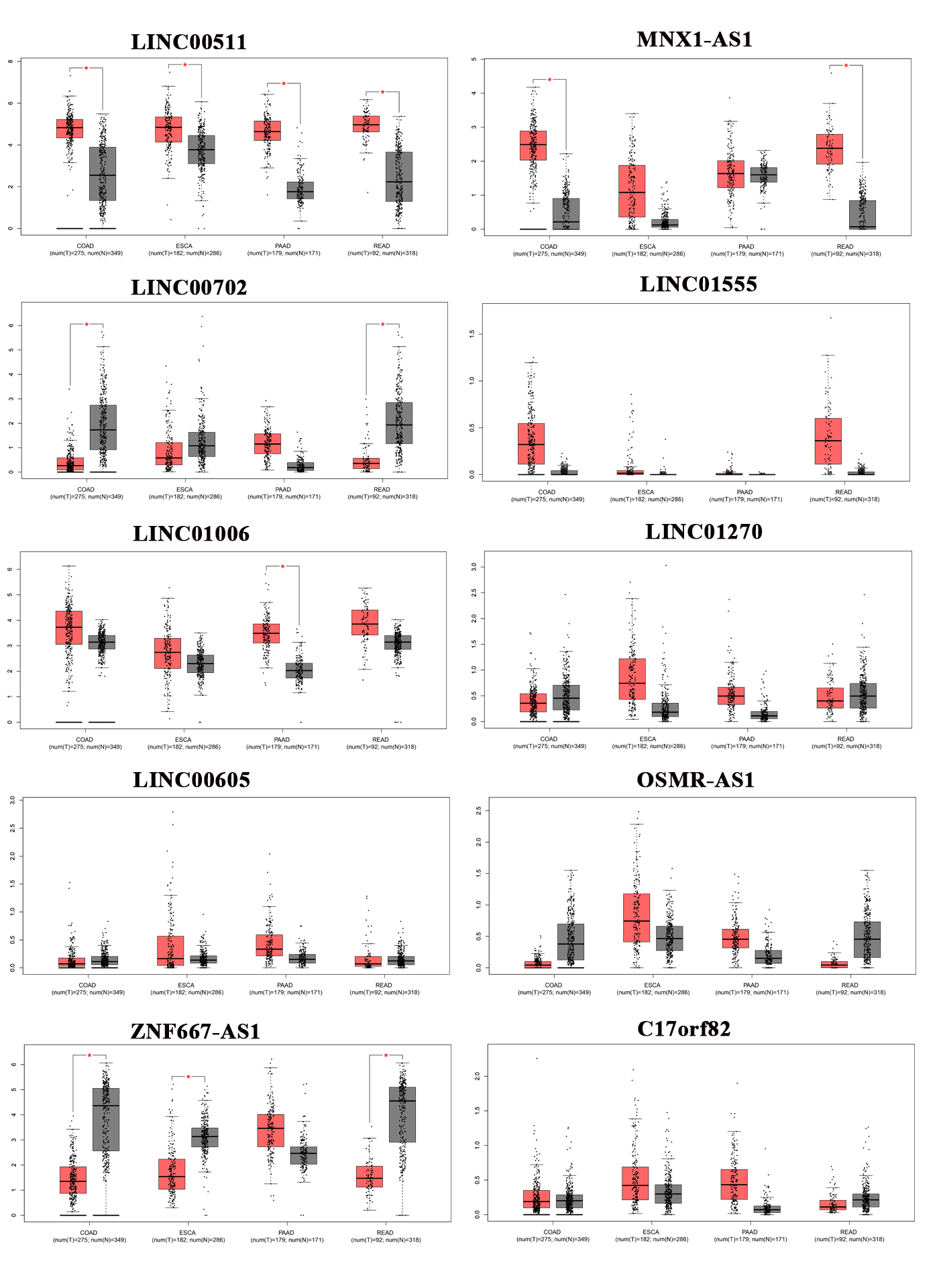

Supplement: Supplementary file 1 — Fig S1 [file JCMM-25-892-s001.tif]
