# Supplementary material for: Analysis of differentially expressed genes in oral epithelial cells infected with Fusobacterium nucleatum for revealing genes associated with oral cancer
Source: J Cell Mol Med. 2020 Dec 2;25(2):892–904. doi: 10.1111/jcmm.16142 (PMC7812288; doi:10.1111/jcmm.16142)
Supplement: Supplementary file 2 — Table S1‐S2 [file JCMM-25-892-s002.docx]

Supplementary Table S1A. Top 20 up-regulated mRNAs and lncRNAs

| mRNA | log2FC | *P*-value | lncRNAs | log2FC | *P-*value |
| --- | --- | --- | --- | --- | --- |
| CNOT6 | 13.41557 | 1.1E-128 | MIR4435-2HG | 5.374798 | 4.15E-14 |
| RPL4 | 13.27071 | 9.3E-117 | AC034213.1 | 4.664954 | 3.89E-31 |
| R3HDM1 | 9.186722 | 2.16E-25 | AL590434.1 | 4.60727 | 1.40E-10 |
| PTPDC1 | 9.161407 | 1.15E-40 | MIATNB | 3.948281 | 1.61E-07 |
| FKTN | 8.908765 | 1.96E-29 | AC129926.1 | 3.8047 | 0.002139 |
| TOPORS | 8.795616 | 3.12E-32 | AP002884.3 | 3.733697 | 2.28E-06 |
| ATP6AP2 | 8.709054 | 2.4E-40 | AC083801.2 | 3.713313 | 5.23E-06 |
| ACTR1A | 8.641493 | 3.72E-21 | PSMB8-AS1 | 3.705218 | 1.51E-06 |
| FRMD5 | 8.5001 | 1.15E-58 | MIR503HG | 3.586239 | 0.00358 |
| PRPSAP2 | 8.149743 | 4.97E-24 | LINC02009 | 3.577998 | 6.24E-34 |
| GCOM1 | 7.710373 | 3.56E-23 | AC133561.2 | 3.450171 | 5.68E-06 |
| OSBPL8 | 7.637827 | 2.1E-23 | AC012555.1 | 3.434179 | 0.000263 |
| RTN3 | 7.604627 | 1.68E-12 | AC007384.1 | 3.417457 | 3.17E-05 |
| FAM83D | 7.590062 | 4.8E-16 | AC073370.1 | 3.406085 | 6.37E-06 |
| GPNMB | 7.56685 | 0.00137 | LINC02537 | 3.378342 | 1.73E-05 |
| WDR1 | 7.262754 | 7.76E-05 | LINC01967 | 3.351598 | 0.000125 |
| CACNB2 | 7.212065 | 1.81E-25 | LINC01607 | 3.344161 | 1.53E-05 |
| ZSCAN4 | 7.17054 | 2.51E-50 | AP003559.1 | 3.321147 | 3.05E-15 |
| ZDHHC20 | 7.063969 | 1.21E-10 | AC130456.2 | 3.285247 | 1.46E-05 |
| AKAP2 | 7.039858 | 0.000031 | AC144450.1 | 3.285221 | 1.48E-05 |

Supplementary Table S1B. Top 20 down-regulated mRNAs and lncRNAs

| mRNA | log2FC | *P*-value | lncRNAs | log2FC | *P*-value | |
| --- | --- | --- | --- | --- | --- | --- |
| LAMA3 | -13.2867 | 2.7E-117 | AC090833.1 | -5.3098 | 3.73E-20 |  |
| CUL4A | -12.2064 | 3.58E-91 | AC245041.2 | -4.62958 | 1.93E-08 |  |
| P4HB | -12.1099 | 4.24E-90 | LINC01224 | -4.44426 | 7.49E-08 |  |
| POLR2B | -12.0756 | 2.34E-87 | LINC01669 | -4.38516 | 1.67E-08 |  |
| NCOA6 | -12.0488 | 8.83E-78 | LINC02407 | -4.38256 | 1.49E-07 |  |
| MAP4 | -11.6065 | 3.93E-81 | LINC00473 | -4.35992 | 0.003366 |  |
| ZYX | -11.5312 | 2.39E-78 | AC092683.1 | -4.32527 | 7.55E-10 |  |
| FUS | -11.3686 | 5.3E-68 | C1orf220 | -4.16259 | 6.33E-07 |  |
| STAG1 | -10.9633 | 1.55E-63 | CU633906.1 | -4.12938 | 4.75E-07 |  |
| GALNT10 | -10.7332 | 4.84E-98 | LINC01060 | -4.03851 | 4.29E-06 |  |
| MYH10 | -10.5665 | 1.9E-64 | AC090124.2 | -4.0202 | 7.75E-08 |  |
| RPL6 | -10.4182 | 2.25E-60 | MIR646HG | -3.83372 | 9.19E-05 |  |
| FBN2 | -10.41 | 4.74E-36 | AP006621.3 | -3.83131 | 7.15E-06 |  |
| CCHCR1 | -10.3372 | 2.98E-59 | LINC01958 | -3.76672 | 5.82E-06 |  |
| FAM83A | -10.1646 | 1.99E-34 | AL354793.1 | -3.72111 | 1.61E-06 |  |
| COL12A1 | -10.0499 | 2.03E-29 | AC091940.1 | -3.66672 | 0.001668 |  |
| DUS2 | -9.98399 | 1.72E-54 | AC090409.2 | -3.64293 | 8.81E-05 |  |
| KIAA1468 | -9.97463 | 2.22E-37 | AC005592.1 | -3.63489 | 8.54E-06 |  |
| ZNF180 | -9.8452 | 2E-51 | LINC00473 | -3.53768 | 1.05E-05 |  |
| ILF3 | -9.80938 | 0.000372 | AL139220.2 | -3.53605 | 1.07E-05 |  |

Supplementary Table S2. Differentially expressed oncogenes and tumor suppressor genes

| Oncogene | log2FC | *P*-value | Oncogene | log2FC | *P*-value |
| --- | --- | --- | --- | --- | --- |
| FAM83D | 7.590062 | 4.8E-16 | AKAP13 | -1.72192 | 2.02E-05 |
| EEF1D | 7.579072 | 1.36E-67 | CAV1 | -1.74845 | 5.16E-06 |
| YY1 | 5.796358 | 7.67E-06 | ETV6 | -1.75258 | 0.001299 |
| MERTK | 5.416381 | 2.23E-16 | MDM2 | -1.78114 | 0.001048 |
| MEFV | 4.958079 | 0.000019 | RPL23 | -1.78873 | 0.000806 |
| MPST | 4.641093 | 6.07E-05 | ILK | -1.81463 | 1.41E-05 |
| PPM1D | 4.409499 | 2.97E-05 | RAB8A | -1.82107 | 0.000011 |
| BAIAP2L1 | 4.310638 | 2.13E-08 | FOSL1 | -1.83034 | 0.000728 |
| PTP4A1 | 4.301009 | 0.000468 | CSF3 | -1.84477 | 1.29E-06 |
| MYB | 4.265538 | 3.53E-05 | COPS3 | -1.8877 | 3.79E-11 |
| EEF1A1 | 4.084128 | 2.84E-05 | PIR | -1.90404 | 7.22E-06 |
| LCK | 3.960284 | 5.61E-07 | CTNNB1 | -1.90926 | 0.003381 |
| CRYAB | 3.841415 | 1.45E-05 | VAV2 | -1.91152 | 1.39E-07 |
| GALNT10 | 3.73523 | 2.5E-51 | DNPH1 | -1.91777 | 0.000663 |
| BOC | 3.085877 | 0.000037 | AKT1 | -1.95526 | 0.00065 |
| DAXX | 3.035323 | 3.88E-15 | PPP2R1A | -2.02237 | 0.002774 |
| SFPQ | 3.035253 | 6.89E-05 | ZBTB16 | -2.07795 | 1.38E-05 |
| FDPS | 2.951658 | 4.23E-05 | ITGA3 | -2.09899 | 1.97E-07 |
| WISP1 | 2.940526 | 0.000116 | CYGB | -2.13369 | 3.97E-15 |
| CUX1 | 2.935772 | 1.78E-06 | CYP24A1 | -2.16917 | 0.001633 |
| BCL6 | 2.932439 | 1.73E-05 | MPST | -2.22144 | 0.00376 |
| SQSTM1 | 2.897838 | 6.85E-77 | PIK3R1 | -2.24602 | 1.03E-05 |
| ASPSCR1 | 2.890188 | 0.001767 | SS18 | -2.32616 | 0.000929 |
| GLI1 | 2.888481 | 9.84E-05 | MECOM | -2.39442 | 0.002511 |
| JAK2 | 2.82247 | 0.001144 | MAML2 | -2.46474 | 0.003253 |
| ARHGEF2 | 2.784818 | 0.000753 | CXCL1 | -2.50813 | 1.7E-60 |
| CUL4A | 2.75699 | 5.69E-19 | SSX2B | -2.5502 | 0.002128 |
| TNFRSF1B | 2.702492 | 2.66E-08 | CREB1 | -2.57605 | 5.58E-13 |
| NFIB | 2.642334 | 0.000593 | RRAS2 | -2.59572 | 0.002634 |
| MKL2 | 2.605142 | 3.29E-05 | FGFR2 | -2.61338 | 0.002489 |
| TINCR | 2.491939 | 7.44E-39 | CBX7 | -2.63978 | 0.001489 |
| TFE3 | 2.45114 | 0.000573 | UBE2C | -2.71146 | 0.001244 |
| ROS1 | 2.425138 | 0.003156 | RHOC | -2.7812 | 0.000715 |
| PLAG1 | 2.413291 | 4.31E-06 | TFCP2 | -2.79544 | 2.61E-07 |
| CREB1 | 2.367947 | 0.002268 | UHRF1 | -2.8587 | 0.000281 |
| MUC1 | 2.311386 | 2.07E-13 | SGK1 | -2.87812 | 0.00049 |
| TBC1D1 | 2.283773 | 1.09E-11 | CABIN1 | -3.15565 | 0.000745 |
| TCF3 | 2.262528 | 0.001754 | PTK7 | -3.17116 | 0.000379 |
| LCN2 | 2.166429 | 2.86E-44 | LMO3 | -3.23833 | 8.71E-05 |
| SALL4 | 2.156413 | 0.000582 | MFNG | -3.30468 | 0.000103 |
| Oncogene | log2FC | *P*-value | Oncogene | log2FC | *P*-value |
| YY1AP1 | 2.15614 | 0.000322 | ELK3 | -3.32381 | 0.000151 |
| NECTIN4 | 2.127779 | 1.04E-20 | IRF2 | -3.34223 | 0.000281 |
| IGF1R | 2.102005 | 0.000529 | SKP2 | -3.43195 | 0.000695 |
| NOV | 2.093424 | 1.08E-50 | HLF | -3.46772 | 0.000107 |
| AURKA | 2.085235 | 6.25E-06 | JUP | -3.52175 | 1.26E-05 |
| YAP1 | 2.059503 | 0.001196 | NUP214 | -3.74406 | 1.99E-05 |
| DDIT3 | 1.998268 | 9.59E-05 | PML | -3.93204 | 5.42E-06 |
| KMT2A | 1.94092 | 7.01E-05 | RINT1 | -3.98103 | 5.57E-07 |
| BANP | 1.918518 | 0.000234 | NFIB | -4.04809 | 7.78E-20 |
| NCOA3 | 1.914133 | 0.0008 | BAX | -4.13661 | 3.02E-05 |
| MST1R | 1.873581 | 0.003763 | SRSF2 | -4.22794 | 8.9E-19 |
| S100A8 | 1.866736 | 3.38E-10 | PDGFB | -4.24442 | 2.13E-08 |
| ATF3 | 1.843686 | 5.39E-05 | URI1 | -4.2845 | 1.01E-05 |
| FOS | 1.835598 | 3.96E-10 | MACC1 | -4.31754 | 0.000874 |
| BIRC3 | 1.820337 | 0.00091 | SETDB1 | -4.35089 | 3.74E-08 |
| SREBF1 | 1.806397 | 0.002413 | IKZF1 | -4.36205 | 2.49E-08 |
| CFLAR | 1.785339 | 0.000414 | NEDD9 | -4.49518 | 0.000451 |
| NPM1 | 1.754663 | 0.001569 | MNAT1 | -4.50972 | 1.04E-05 |
| PTK7 | 1.7424 | 8.73E-05 | HMGA2 | -4.59836 | 2.51E-06 |
| FYN | 1.730513 | 2.58E-06 | TAZ | -4.67455 | 3.28E-06 |
| PSIP1 | 1.649108 | 0.001083 | GPM6B | -4.69599 | 2.74E-13 |
| HMGA2 | 1.632776 | 1.24E-12 | KDM5B | -4.88055 | 3.65E-05 |
| TPD52 | 1.627231 | 0.000041 | AHI1 | -4.9226 | 3.95E-39 |
| BCL3 | 1.5913 | 2.33E-07 | DCUN1D1 | -5.3973 | 0.00013 |
| SNAI1 | 1.585766 | 1.72E-24 | SET | -5.47365 | 1.64E-10 |
| PDZK1IP1 | 1.534274 | 0.002687 | CDK4 | -5.87754 | 8.45E-10 |
| HNRNPA1 | -1.50218 | 2.94E-18 | BMI1 | -6.05147 | 2.39E-15 |
| EGFR | -1.51091 | 3.99E-13 | RAF1 | -7.18307 | 2.72E-18 |
| LDHB | -1.57569 | 4.35E-05 | DAXX | -7.29829 | 1.81E-11 |
| FOXP1 | -1.59654 | 0.001729 | ETV1 | -7.9026 | 1.36E-24 |
| PTP4A1 | -1.60727 | 6.73E-06 | EWSR1 | -8.44097 | 9.07E-16 |
| GNAS | -1.62225 | 4.38E-05 | GALNT10 | -10.7332 | 4.84E-98 |
| AXL | -1.63832 | 7.06E-18 | CUL4A | -12.2064 | 3.58E-91 |
| FNDC3B | -1.66533 | 0.002529 |  |  |  |

| TSGs | log2FC | *P*-value | TSGs | | log2FC | *P*-value |
| --- | --- | --- | --- | --- | --- | --- |
| TOPORS | 8.795616 | 3.12E-32 | | PDCD4 | -4.91701 | 0.000216 |
| TNFRSF12A | 6.75849 | 3.08E-22 | | IGFBP3 | -4.8691 | 4.28E-05 |
| LHX6 | 6.438498 | 7.95E-08 | | BRMS1 | -4.74088 | 2.69E-08 |
| BCL2L11 | 6.010223 | 0.00049 | | ZMYND11 | -4.67206 | 0.000545 |
| HIVEP1 | 5.982908 | 9.58E-22 | | EZH1 | -4.591 | 0.000431 |
| BRSK1 | 5.876965 | 2.7E-12 | | CDK2AP1 | -4.4363 | 0.000244 |
| ZMYND11 | 5.803602 | 0.000141 | | MBD4 | -4.36313 | 6.22E-08 |
| PRODH | 4.966911 | 0.001648 | | IKZF1 | -4.36205 | 2.49E-08 |
| POU2F3 | 4.767489 | 8.19E-11 | | NBN | -4.3352 | 8.75E-08 |
| NRCAM | 4.516627 | 0.00267 | | IKZF2 | -4.28072 | 7.69E-08 |
| PTPN2 | 4.392721 | 4.61E-06 | | KCNRG | -4.15675 | 0.002019 |
| FAM172A | 4.371623 | 5.9E-08 | | BAX | -4.13661 | 3.02E-05 |
| HSPD1 | 4.28559 | 0.000019 | | ING5 | -4.05932 | 1.59E-08 |
| EEF1A1 | 4.084128 | 2.84E-05 | | RINT1 | -3.98103 | 5.57E-07 |
| BTG3 | 3.996278 | 6.75E-10 | | MTSS1 | -3.95605 | 0.000365 |
| CMTM3 | 3.978182 | 0.000055 | | PML | -3.93204 | 5.42E-06 |
| PYHIN1 | 3.975204 | 0.00116 | | PPP2CB | -3.80152 | 1.05E-06 |
| GADD45B | 3.970716 | 2.67E-07 | | RBM5 | -3.79452 | 0.000023 |
| GSN | 3.887041 | 2.2E-06 | | CCNDBP1 | -3.78845 | 3.71E-06 |
| TCF4 | 3.850211 | 0.003507 | | PLA2R1 | -3.73305 | 0.00002 |
| MBD4 | 3.697679 | 9.01E-11 | | PPP2R1B | -3.70002 | 0.000485 |
| PLA2R1 | 3.634792 | 0.00365 | | VEZT | -3.66845 | 9.16E-06 |
| EYA4 | 3.617362 | 6.36E-07 | | PPP2R5C | -3.57761 | 0.000188 |
| ZYX | 3.503618 | 3.49E-06 | | MAP3K4 | -3.5349 | 6.34E-06 |
| BIN1 | 3.476263 | 0.000597 | | ZNF185 | -3.52501 | 0.000032 |
| ANXA7 | 3.407256 | 0.001707 | | JUP | -3.52175 | 1.26E-05 |
| SMARCB1 | 3.353323 | 0.002171 | | DAB2 | -3.49665 | 3.99E-05 |
| BBC3 | 3.342341 | 0.000402 | | ARNTL | -3.47476 | 0.00361 |
| BMF | 3.329881 | 0.001364 | | TSC22D1 | -3.43918 | 0.003592 |
| NDRG2 | 3.261667 | 1.61E-05 | | SKP2 | -3.43195 | 0.000695 |
| PPARA | 3.253825 | 8.09E-06 | | CNOT3 | -3.39761 | 6.8E-08 |
| GORAB | 3.112565 | 0.000638 | | MDC1 | -3.35507 | 0.000126 |
| DAPK3 | 3.109104 | 0.001278 | | ARID1A | -3.3325 | 0.002777 |
| KRIT1 | 3.106243 | 2.33E-05 | | RASAL1 | -3.31502 | 0.000134 |
| NUPR1 | 3.057456 | 1.85E-12 | | SDHA | -3.30781 | 2.44E-05 |
| LITAF | 2.999763 | 0.000286 | | KMT2C | -3.29326 | 5.28E-07 |
| GANAB | 2.976603 | 0.000128 | | CIZ1 | -3.28543 | 1.75E-05 |
| CUX1 | 2.935772 | 1.78E-06 | | PRDM2 | -3.28134 | 0.000057 |
| CREM | -2.49215 | 0.003359 | | WWOX | -3.2503 | 7.89E-12 |
| GLI1 | 2.888481 | 9.84E-05 | | HTRA2 | -3.24042 | 0.002069 |
| CD82 | 2.876569 | 0.000187 | | MARVELD1 | -3.16906 | 9.96E-05 |
| ACY1 | 2.841838 | 0.000462 | | AHRR | -3.16906 | 9.96E-05 |
| KCNRG | 2.784623 | 4.62E-05 | | STK10 | -3.16586 | 6.86E-07 |
| TSGs | log2FC | *P*-value | | TSGs | log2FC | *P*-value |
| DPH1 | 2.676812 | 9.01E-06 | | CBFA2T3 | -3.06696 | 0.000388 |
| PCDH9 | 2.629441 | 3.33E-05 | | KDM6A | -3.02326 | 8.32E-12 |
| PKD1 | 2.607394 | 0.000194 | | SLC39A4 | -2.92294 | 4.49E-09 |
| EXTL1 | 2.571155 | 0.001107 | | ALDH1A2 | -2.88059 | 0.001475 |
| BMPR1A | 2.498172 | 0.001261 | | MFSD2A | -2.84139 | 0.003656 |
| AMH | 2.486132 | 0.001598 | | CLU | -2.82561 | 1.6E-06 |
| HLTF | 2.453622 | 0.001213 | | MAPK10 | -2.81967 | 0.000919 |
| TNFSF9 | 2.416315 | 1.09E-05 | | EGLN3 | -2.80282 | 0.000867 |
| PHB | 2.383394 | 0.002423 | | PPM1A | -2.74815 | 0.000276 |
| SLC39A1 | 2.37632 | 0.000657 | | EHF | -2.71448 | 1.13E-21 |
| NRSN2 | 2.348791 | 0.000201 | | SAMD9L | -2.69904 | 0.001055 |
| PEA15 | 2.316318 | 2.24E-09 | | SMARCA2 | -2.6464 | 0.000262 |
| EPHB3 | 2.266471 | 0.000136 | | CDH4 | -2.57071 | 0.002212 |
| TCF3 | 2.262528 | 0.001754 | | RPS6KA2 | -2.54091 | 0.002869 |
| STRADA | 2.252002 | 4.77E-05 | | HDAC3 | -2.52912 | 0.002244 |
| BRD7 | 2.238524 | 0.002867 | | NF2 | -2.52233 | 0.002898 |
| INTS6 | 2.236959 | 1.46E-20 | | LRIG3 | -2.51295 | 0.001778 |
| HINT1 | 2.215292 | 0.000647 | | ST7 | -2.49284 | 0.000571 |
| IL17RD | 2.193561 | 0.002857 | | CREM | -2.49215 | 0.003359 |
| APC | 2.174831 | 0.00184 | | STRADA | -2.46987 | 0.000542 |
| HSPB7 | 2.16706 | 1.28E-08 | | DKK1 | -2.46903 | 5.71E-10 |
| RAD23B | 2.16521 | 2.82E-05 | | RBM4 | -2.40101 | 0.002372 |
| CYB561D2 | 2.156525 | 0.003635 | | IRF1 | -2.33024 | 5.11E-08 |
| SALL4 | 2.156413 | 0.000582 | | CAMTA1 | -2.19454 | 0.00228 |
| WDR11 | 2.141528 | 0.003118 | | CNDP2 | -2.19144 | 0.002287 |
| CADM1 | 2.09772 | 0.000518 | | CYGB | -2.13369 | 3.97E-15 |
| NOV | 2.093424 | 1.08E-50 | | ABCG2 | -2.10475 | 4.1E-08 |
| FOXO6 | 2.072457 | 6.49E-07 | | GADD45B | -2.09897 | 2.68E-14 |
| YAP1 | 2.059503 | 0.001196 | | EXT2 | -2.09244 | 0.000107 |
| PRDM1 | 2.058259 | 0.001481 | | OSCP1 | -2.0901 | 0.003664 |
| CDKN1C | 1.997358 | 0.003246 | | CASP2 | -2.07905 | 1.44E-05 |
| BTG2 | 1.945582 | 0.000035 | | ZBTB16 | -2.07795 | 1.38E-05 |
| FLCN | 1.933455 | 0.000192 | | TGFBR2 | -2.05073 | 2E-119 |
| BANP | 1.918518 | 0.000234 | | PEA15 | -2.03742 | 3.7E-07 |
| MST1R | 1.873581 | 0.003763 | | MT2A | -2.00473 | 1.83E-14 |
| PHF6 | 1.872195 | 6.47E-08 | | DFFA | -1.98355 | 0.002645 |
| VEGFA | 1.864676 | 2.23E-09 | | S100A2 | -1.97373 | 8.43E-09 |
| CBFA2T3 | 1.852907 | 0.000787 | | KRIT1 | -1.96406 | 2.69E-06 |
| ATF3 | 1.843686 | 5.39E-05 | | CSNK1A1 | -1.92471 | 0.00092 |
| NOTCH3 | 1.815348 | 2.35E-49 | | ITGB1 | -1.90938 | 0.000412 |
| NDRG1 | 1.782459 | 0.002038 | | TCF7L2 | -1.90206 | 0.001298 |
| PRDM2 | 1.766372 | 0.000542 | | PHB | -1.85565 | 0.000639 |
| NPM1 | 1.754663 | 0.001569 | | TCHP | -1.84826 | 0.001286 |
| TSGs | log2FC | *P*-value | | TSGs | log2FC | *P*-value |
| GBP1 | 1.74953 | 0.000538 | | ILK | -1.81463 | 1.41E-05 |
| NR4A1 | 1.733707 | 0.002822 | | NRF1 | -1.7833 | 0.001234 |
| ATM | 1.721551 | 0.002407 | | DNAJB1 | -1.77573 | 3.41E-05 |
| EGLN3 | 1.642301 | 0.001022 | | ETV6 | -1.75258 | 0.001299 |
| HBP1 | 1.561047 | 1.01E-05 | | CAV1 | -1.74845 | 5.16E-06 |
| BLNK | 1.541152 | 0.000791 | | HINT1 | -1.70768 | 1.6E-07 |
| EGR2 | 1.539738 | 1.85E-07 | | XRCC5 | -1.7038 | 0.000233 |
| PKNOX1 | 1.527902 | 0.002362 | | CITED2 | -1.66148 | 3.54E-33 |
| SHQ1 | 1.52187 | 2.82E-06 | | DLG1 | -1.65247 | 0.002482 |
| ZMYND10 | 1.520068 | 3.17E-05 | | FBXO31 | -1.6493 | 0.00023 |
| DICER1 | 1.51619 | 0.001819 | | SPRY4 | -1.63852 | 1.77E-05 |
| ZYX | -11.5312 | 2.39E-78 | | TSC1 | -1.63366 | 0.001515 |
| FAT1 | -8.2663 | 1.78E-10 | | PTPRK | -1.62811 | 0.000625 |
| SEMA3F | -8.24128 | 1.03E-06 | | GNMT | -1.62287 | 0.002046 |
| HSPD1 | -8.20904 | 1.33E-16 | | PBRM1 | -1.60197 | 2.94E-53 |
| CEACAM1 | -7.60702 | 2.32E-20 | | RHOBTB2 | -1.60197 | 2.94E-53 |
| CASP8 | -7.39231 | 1.44E-12 | | AHNAK | -1.60184 | 0.000962 |
| DUSP6 | -7.29105 | 1.94E-20 | | TET2 | -1.59847 | 0.000531 |
| PRICKLE1 | -7.21501 | 2.31E-22 | | FOXP1 | -1.59654 | 0.001729 |
| BRCA1 | -7.07452 | 4.74E-21 | | LIMA1 | -1.59121 | 0.003709 |
| DNAJC11 | -6.944 | 7.3E-16 | | DDX58 | -1.56623 | 1.66E-05 |
| MEN1 | -6.88456 | 0.000434 | | CXXC4 | -1.54576 | 4.65E-06 |
| PYCARD | -6.08079 | 7.63E-16 | | IFT88 | -1.53961 | 2.77E-06 |
| BIN1 | -5.84564 | 0.000181 | | MAX | -1.53353 | 0.001753 |
| BRF1 | -5.52517 | 1.01E-13 | | INTS6 | -1.5326 | 2.85E-24 |
| DOK1 | -5.4987 | 1.94E-06 | | ING3 | -1.52555 | 5.17E-06 |
| PRKAR1A | -5.13495 | 4.45E-07 | | PLK2 | -1.52406 | 4.7E-10 |
| TANK | -5.10686 | 9.07E-11 | | GORAB | -1.51764 | 0.000113 |
| SMAD4 | -5.0125 | 3.39E-07 | | BRCA2 | -1.511 | 0.000195 |
| TCF4 | -4.9327 | 1.23E-10 | |  |  |  |

Abbreviation

TSGs: tumor suppressor genes
